# Barriers and strategies for cervical cancer screening: What do female university students know and want?

**DOI:** 10.1371/journal.pone.0257529

**Published:** 2021-10-05

**Authors:** Hye Young Shin, Soo Yeon Song, Jae Kwan Jun, Ka Young Kim, Purum Kang

**Affiliations:** 1 National Cancer Control Institute, National Cancer Center, Goyang, Republic of Korea; 2 College of Nursing, Baekseok Culture University, Cheonan-si, Republic of Korea; 3 Graduate School of Cancer Science and Policy, National Cancer Center, Goyang, Republic of Korea; 4 Department of Nursing, College of Nursing, Gachon University, Incheon, Republic of Korea; 5 College of Nursing, Woosuk University, Wanju, Jeollabuk-do, Republic of Korea; Ordu University, TURKEY

## Abstract

**Objective:**

This study aimed to identify the distinct barriers and knowledge level of cervical cancer screening among female university students and establish intervention strategies to overcome these barriers.

**Methods:**

This study used a mixed-methods design with 26 female university students aged 20–29 years. We first conducted a quantitative online survey for the same study participants, divided them into three groups, and conducted focus group interviews (FGIs). Group A: participants who had sexual experience and had undergone cervical cancer screening; Group B: participants who had sexual experience and had not undergone cervical cancer screening; Group C: participants who did not have sexual experience and had not undergone cervical cancer screening.

**Results:**

The participants’ ages were 21.92 ± 1.26 years. The knowledge levels for cervical cancer and screening were low to moderate. The four main themes that emerged as barriers to cervical cancer screening through the FGIs were: 1) socio-cultural barrier: conservative social perception of unmarried women’s sexual life, 2) knowledge barrier: lack of knowledge and information, 3) psychological barrier: discomfort, and 4) practical barrier: time-consuming. The three themes identified for strategies were: 1) socio-cultural intervention: changing social perceptions and ensuring confidentiality, 2) educational intervention: improvement of knowledge and accessibility, and 3) alternative screening intervention: comfortable screening methods.

**Conclusions:**

While university students’ sexual experience rapidly increased, the socio-cultural perceptions of sexual health remained closed, and they had a reasonably low level of knowledge about cervical cancer screening. Therefore, various strategies sensitive to female university students’ culture should be implemented to increase the knowledge level, and social efforts should be made to change the socio-cultural perception of unmarried young women’s sexual health.

## Introduction

Globally, the age-standardized incidence rate for cervical cancer is estimated at 13.1/100,000 individuals, and the rate is high in developing countries [1]. In Korea, the incidence rate of cervical cancer was 8.7/100,000 individuals in 2017, and it had significantly decreased by 3.1% annually from 2007 to 2017 [2]. However, a significant increasing trend in cervical cancer has been observed in young women aged 20–29 years [3]. Moreover, the incidence rate of cervical cancer in women aged 15–34 years ranked third in the incidence rates of all cancers, whereas it ranked seventh among all women [2].

The Papanicolaou test (Pap test) is a promising cervical cancer screening method to detect cervical cancer early and decrease invasive cervical cancer. In developed countries, cervical cancer screening rates in young women in their 20s were approximately 60%–70%, [4, 5] but in Korea, it is much lower, at approximately 20% [6]. In particular, female university students’ cervical cancer screening rate in their early 20s was approximately 5–8% [7, 8]. Meanwhile, the National Cancer Screening Program (NCSP) in Korea has included women in their 20s as the target population for cervical cancer screening since 2016 [6]. As per this system, the target population is notified of their eligibility for screening every two years via mail. After undergoing the screening, the test results are provided via mail or a mobile phone text message, depending on their preference. To date, even though target women in their 20s have received at least two mails since 2016, there has not been an apparent increase in the cervical cancer screening rate [6].

A distinct barrier to cervical cancer screening includes psychological factors related to testing procedure associated with exposure of the female genital [9, 10]. Along with this, it has been reported that awareness and knowledge on the importance and purpose of cervical cancer screening lack among young women [11]. However, despite the continued low cervical cancer screening rate in young women, few studies have been conducted to develop strategies to overcome these barriers by in-depth analysis of the barriers to cervical cancer screening among them.

Therefore, this study aimed to identify the distinct barriers and knowledge level of cervical cancer screening among female university students and establish intervention strategies to overcome these barriers.

## Methods

### Study design

This study adopted a mixed-methods design. The quantitative method was conducted prior to the FGIs through an online survey. It aimed to acquire responses on personal information, sexual experience, and the knowledge level of cervical cancer screening by guaranteeing individual anonymity in this study, along with a closed sex culture in Korea [11]. The qualitative method used FGIs to identify the barriers and strategies for cervical cancer screening.

### Study participants and data collection

To recruit study participants, we used the snowball sampling recruitment technique and the method of posting a recruitment notice on the online community bulletin board of a metropolitan area’s university. The inclusion criteria comprised female university students aged 20–29 years with no history of cervical cancer. Study participants who were assigned to either of the three groups: Group A, participants who had sexual experiences and had undergone cervical cancer screening; Group B: participants who had sexual experiences but had not undergone cervical cancer screening; Group C, participants who had no sexual experience and had not undergone cervical cancer screening. The research staff received written informed consent prior to the FGI. This study was approved by the Institute of Research Board of Baekseok Culture University (2-7008132-A-N-01).

#### Quantitative method

As a quantitative method, we conducted an online survey using the same FGI participants. The survey questionnaire included questions on socio-demographic characteristics, including age, university major, and grade. In addition, questions on sexual experiences and cervical cancer screening history; knowledge of prevention (3 items), risk factors (4 items), and symptoms (4 items) of cervical cancer; and knowledge of the national cervical cancer screening program (3 items) were included.

#### Qualitative method

Based on the recommended sample size for an FGI [12], 7–11 female students participated in each focus group (see Table 1). During the FGI, open-ended questionnaires were used as an interview guide that asked about knowledge of cervical cancer and screening, information acquisition channels, and barriers to and facilitators of cervical cancer screening. We emailed the FGI questionnaires to study participants in advance. An experienced moderator, a registered nurse, led all FGIs, and each interview lasted for approximately 90–120 minutes. The participants were identified using pseudonyms.

**Table 1:** Participants’ general characteristics (N = 26).

Characteristics	Total		Group A(n = 7)		Group B(n = 11)		Group C (n = 8)		*p-value*
Age, mean (SD)	21.92	(1.26)	22.30	(1.11)	22.40	(1.21)	21.00	(1.07)	0.038
Major, n (%)									
Humanities and social sciences/ arts	17	(65.4)	6	(85.7)	4	(36.4)	7	(87.5)	0.006
Natural sciences/ Engineering	9	(34.6)	1	(14.3)	7	(63.6)	1	(12.5)	
Grade, n (%)									
Sophomore	2	(7.7)	0	(0.0)	0	(0.0)	2	(25.0)	0.001
Junior	16	(61.5)	5	(71.4)	5	(45.5)	6	(61.5)	
Senior	8	(30.8)	2	(28.6)	6	(54.6)	0	(0.0)	
Knowledge, mean (SD)									
Prevention (possible range: 0–3)	1.62	(0.57)	1.86	(0.38)	1.55	(0.69)	1.50	(0.53)	0.435
Risk factors (possible range: 0–4)	2.58	(0.86)	3.14	(0.38)	2.27	(0.90)	2.50	(0.93)	0.102
Symptoms (possible range: 0–4)	2.50	(1.03)	2.71	(1.11)	2.55	(0.82)	2.25	(1.28)	0.689
Knowledge for national cervical cancer screening program	1.35	(0.80)	2.00	(0.00)	1.27	(0.90)	0.88	(0.64)	0.016
(possible range: 0–3), mean (SD)									

### Data analysis

We used descriptive statistics using frequencies and percentages, or means and standard deviations for analyzing the participants’ socio-demographic backgrounds and knowledge levels. The FGI data was analyzed using conventional content analysis for inductive category development [13], wherein three coders initially read the transcripts repeatedly and independently to derive codes, discussed discrepancies of the emerged codes, and achieved the best coding. Moreover, coders sorted these codes into subthemes, which they used to identify main themes. Finally, the research team repeatedly discussed the main themes and subthemes to encompass the phenomenon.

## Results

A total of 26 identical participants were included in both the online survey and FGI analysis. Their demographic characteristics are presented in Table 1. The participants’ mean age was 21.92 ± 1.26 years. Approximately 65.4% of the participants were from the humanities and social sciences/arts, followed by 34.6% from natural sciences/engineering. The knowledge level of cervical cancer was moderate, but the knowledge level of the cervical cancer screening program implemented in the government was low (1.35 points [0–3]).

### Main themes over barriers to cervical cancer screening

The following four themes were identified: socio-cultural barrier, knowledge barrier, psychological barrier, and practical barrier. A summary of the subthemes and relevant quotes is also presented (Table 2).

**Table 2:** Barriers to participation in cervical cancer screening (N = 26).

Groups			Themes
A	B	C	
			**Theme 1. Socio-cultural barrier: conservative social perception of unmarried women’s sexual life**
**M**	**M**	**M**	**1) Negative social view about unmarried young women’s sexual life**
			“My mom does not know anything about HPV vaccination or cervical cancer screening. If I told her that I was getting a screening done, she would view me negatively.”
			“My family thinks that their daughter has never had sex before.”
			“I would be kicked out of my parents’ house if they heard that I had got screened for cervical cancer.”
			“People think that only people with sexual experience visit an obstetrics and gynecology.”
**M**	**M**	**N**	**2) Fear of social stigma about sexual experience**
			“I am afraid that in the year-end tax, there will be a gynecology medical record. Through this recode, my mom may be aware of my visit to an obstetrics and gynecology, and sexual experience.”
			“I just want to pay every time to get cervical cancer screening (opportunistic screening) because I am afraid that the screening result will be sent home by mail, and my parents will know that I have been checked.”
			“If the result is abnormal, I am afraid its record would remain for a long time.”
			**Theme 2. Knowledge barrier: lack of knowledge and information**
**N**	**S**	**M**	**1) Misunderstanding of cervical cancer screening associated with HPV vaccination**
			“I recently learned that the country was offering free cervical cancer screening, but since I was vaccinated, I thought I would not get cervical cancer.”
			“The screening that costs approximately 200,000 won (200$) is a high for me.” (the cost means vaccination)
**S**	**M**	**M**	**2) Insufficient information**
			“The hospitals’ websites do not provide detailed cervical cancer screening procedure and methods, and most of them promote hospitals.”
			“I think I am less likely to get cancer because I am young.”
			“I am not familiar with the procedure about cervical cancer screening, so I think the vague fear of the screening is the biggest.”
			**Theme 3. Psychological barrier: discomfort**
**M**	**M**	**M**	**1) Uncomfortable with the test procedure**
			“I had pain when I was tested and also had a weird feeling.”
			“When I looked up reviews on cervical cancer screening, most of the reviews said that the screening process was a shame.”
**M**	**M**	**M**	**2) Reluctance to opposite sex doctor**
			“There was only a male doctor in the clinic… I was examined behind the curtains, but I felt weird and awful.”
			“I am reluctant to get tested if the doctor is a man.”
			“If I needed to be examined, I would get examined, but I am most worried about the gender of the doctor.”
			**Theme 4. Practical barrier: time-consuming**
**M**	**M**	**N**	**1) Lack of time**
			“I had to wait a long time to see a female doctor at the clinic, but the treatment time was very short.”
			“Actually, I did not have the time.”

HPV, human papillomavirus.

Group A: Participants who had sexual experience and had undergone cervical cancer screening; Group B: Participants who had sexual experience and had not undergone cervical cancer screening; Group C: Participants who did not have sexual experience and had not undergone cervical cancer screening.

**M**, the subtheme emerged in most of the participants; **S,** the subtheme emerged in some of the participants; **N,** the subtheme emerged in none of the participants.

#### Theme 1. Socio-cultural barrier: Conservative social perception of unmarried women’s sexual life

Two subthemes were identified under this theme. First, a negative social view about unmarried young women’s sexual life was derived from all three groups, particularly groups A and B (Table 2). The perception of parents was the biggest obstacle to cervical cancer screening as well as that of the surrounding people. Most of the participants said that their parents were unaware of their sexual experience and would be negative about them if they knew it. As an example, the response from a participant in group A is reproduced below.

*“My mom does not know anything about human papillomavirus (HPV) vaccination or cervical cancer screening, and if I told her that I was getting a screening done, she would view me negatively.”*

Second, in a similar context, fear of social stigma about the sexual experience was drawn from groups A and B. Most participants were concerned that the screening records would be known to someone (especially their parents and the insurance company). A woman commented:

*“I am afraid that in the year-end tax, there will be a gynecology medical record. Through this recode, my mom may be aware of my visit to an obstetrics and gynecology, and sexual experience.”*

*“If the result is abnormal, I am afraid that its record would remain for a long time.”*

#### Theme 2. Knowledge barrier: Lack of knowledge and information

In the subthemes, misunderstanding of cervical cancer screening associated with HPV vaccination, as well as insufficient information about the screening, were identified (Table 2). Except for some participants in group A, who had undergone cervical cancer screening, a lack of knowledge about cervical cancer screening was evident in both B and C groups. A few women said the following:

*“I recently learned that the country has been offering free cervical cancer screening, but since I was vaccinated, I thought I would not develop cervical cancer.”*

*“The screening that costs approximately 200,000 won (200$) is high for me.” (Here, the cost means vaccination)*

In the subtheme of limited information about the screening, participants in all three groups said it was difficult to find practical information about hospital appointments or get screening from the hospitals’ websites or screening-related websites.

#### Theme 3. Psychological barrier: Discomfort

Psychological distress related to being uncomfortable with the test and reluctance to procedure conducted by male doctors was identified (Table 2). Regardless of whether they were screened, the three groups expressed that they were distressed, ashamed, and afraid of cervical cancer screening. The majority of participants had a strong aversion to male doctors. One participant stated:

*“There was only a male doctor in the clinic…I was examined behind curtains, but I felt weird and awful.”*

#### Theme 4. Practical barrier: Time-consuming

A lack of time was identified in both groups A and B (Table 2). Some women complained about long waiting times at hospitals for female doctors and no personal time. Some participants said the following:

*“I had to wait a long time to see a female doctor at the clinic, but the treatment time was very short.”*

*“Actually, I did not have time.”*

### Main themes over strategies for cervical cancer screening

Three themes were identified: socio-cultural intervention, educational intervention, and alternative screening intervention. A summary of the subthemes and relevant quotes is presented in Table 3.

**Table 3:** Strategies for participation in cervical cancer screening (N = 26).

Groups			Themes
A	B	C	
			**Theme 1. Socio-cultural intervention: changing social perceptions and ensuring confidentiality**
**M**	**M**	**M**	**1) Change of a negative social view**
			“I think we first need to recognize that cervical cancer screening is just a test of our bodies. If we change our perceptions, we will naturally be interested in the screening.”
			“My mom said, ‘I received a mail for you to be examined, but you do not need it, do you?’ This does not appear to be the correct approach. Therefore, I think it is necessary to educate parents.”
**M**	**M**	**N**	**2) Guarantee of anonymity**
			“It would be nice if only I could see the results on the web (to avoid parents’ attention).”
			“Creating and distributing handouts on counseling or setting up professional counseling centers will ensure anonymity and help answer questions.”
			**Theme 2. Educational intervention: improvement of knowledge and accessibility**
**M**	**M**	**M**	**1) Peer educator**
			“After all my friends had listened to my story, they told me that they would be examined and vaccinated.”
			“Rather than being influenced by information that cervical cancer screening is really dangerous, I think I will get screened when my friend asks me to try it.”
			“There was little awareness of cervical cancer, but I was most affected by my friends.”
**M**	**M**	**M**	**2) Online communication and information**
			“If the information on cervical cancer screening comprises online videos posted on YouTube, friends will know about it.”
			“If the government sends a mobile text message to inform the screening recipients that they are the target recipients of the cervical cancer screening and the URL for detailed screening information, they will be able to access the screening easily.”
			“It would be great if information about cervical cancer screening was posted in the school campus community.”
			“There is an online channel run by three YouTubers that is more reliable, and I think it is better to view the videos made by these professional YouTubers than to read its written account.”
**M**	**S**	**M**	**3) Easy and straightforward description**
			“I think it is essential to convey not only terms that doctors know, but also words that are common to the general public.”
			“In fact, the national website includes too much text and professional content. . . but, to keep people’s attention, it would be better if the information is short, simple, and easy to see.”
**M**	**M**	**M**	**4) Detail information for initial screening**
			“Young women need the detailed information to ward off cervical cancer even if they have no symptoms.”
			“If a lot of information about the screening process is provided, young people will have fewer negative thoughts about the screening.”
			**Theme 3. Alternative screening intervention: comfortable screening methods**
**M**	**M**	**N**	“I would prefer drawing blood for the screening method or something like that… I could then do it without hesitation since it would not be very much of a shame.”
			“I would rather have a better way to draw blood.”

Group A: Participants who had sexual experience and had undergone cervical cancer screening; Group B: Participants who had sexual experience and had not undergone cervical cancer screening; Group C: Participants who did not have any sexual experience and had not undergone cervical cancer screening.

**M**, the subtheme emerged in most of the participants; **S,** the subtheme emerged in some of the participants; **N,** the subtheme emerged in none of the participants.

#### Theme 1. Socio-cultural intervention: Changing social perceptions and ensuring confidentiality

In all the groups, a majority of the participants expressed that gender-based social perceptions of women were obstructive, and some participants spoke of the necessity of sex education, especially for parents, to change their social perceptions (Table 3). In reality, groups A and B, who were more interested in screening, emphasized that strategies for ensuring confidentiality about privacy issues should be established.

*“It would be nice if only I could see the results on the web (to avoid parents’ attention).” “Creating and distributing handouts on counseling or setting up professional counseling centers will ensure anonymity and help answer questions.”*

#### Theme 2. Educational intervention: Improvement of knowledge and accessibility

Several strategies for improving the knowledge that emerged in the various sub-themes are listed in Table 3. Most of the participants expressed having been affected by recommendations or information on cervical cancer screening received from friends. One woman even remarked that she would have been more influential if her friend had majored in nursing. In addition, some women said the following:

*“After all my friends had listened to my story, they told me that they would be examined and vaccinated.”*

*“Rather than being influenced by information that cervical cancer screening is really dangerous, I think I will get screened when my friend asks me to try it.”*

Online communication and information (YouTube, online campus community, etc.) that increase participants’ accessibility have also been discussed. Another approach to accessibility was to use easy and straightforward descriptions rather than professional terms for cervical cancer screening. Moreover, given that university women having little awareness of cervical cancer and being in the first screening, the need for detailed information on threats posed by cervical cancer and the screening procedure were discussed. Some women commented:

*“If the information on cervical cancer screening comprises online videos posted on YouTube, friends will know about it.”*

*“I think it is essential to convey not only terms that doctors know, but also words that are common to the general public.”*

*“Young women need the detailed information to ward off cervical cancer even if they have no symptoms.”*

#### Theme 3. Alternative screening intervention: Comfortable screening methods

Some participants eligible for cervical cancer screening with sexual experience, with or without screening experience, expressed the need for alternative methods such as blood tests, mainly for emotional reasons listed in Table 3.

*“I would prefer drawing blood for the screening method or something like that…I could then do it without hesitation since it would not be very much of a shame.”*

*“I would rather have a better way to draw blood.”*

## Discussion

This study identified low to moderate level of knowledge of cervical cancer screening among female university students through a quantitative survey. We derived the distinctive barriers to receiving cervical cancer screening and tailored strategies to overcome these barriers through FGIs. The four key themes for the screening barriers were socio-cultural, knowledge, psychological, and practical barrier, and the following three key themes emerged for screening strategies: socio-cultural, educational, and alternative screening intervention.

Interestingly, the negative social view of the sexual life of unmarried young women was evident in all focus groups, which might be closely related to Korea’s Confucian culture that espouses male-centered patriarchy, with the female gender being much more closed than the male gender [14]. In our findings, sexual activity among unmarried young women generally carried stigma and shame, which was aligned with the results of a previous study [11]. Also, our study confirmed that mothers’ conservative attitude toward the sexual life of their daughters had a great influence on preventing the participants from being screened for cervical cancer. A majority of the participants expressed the fear that their mothers might know they have been screened through screening results received by postal mail or medical records at the year-end tax. Likewise, a Korean study analyzing parents’ perspectives on having their daughters screened for cervical cancer reported that parents rarely recognized the need for their daughters’ screening [15]. The closed social awareness related to the sexual life of young women has often been depicted in several Eastern countries with cultures similar to Korea [16, 17]. Meanwhile, it has been reported that a culturally approached education or culturally driven motivational intervention might be influential in increasing cancer screening [18, 19]. In our study, socio-cultural strategies to improve social awareness were also discussed. First, the need for education on women’s perception of the importance of their body, and in particular, education on parents’ perception of their daughters’ sexual health, was insisted. Second, as an accessible method within the current socio-cultural context, strategies for ensuring anonymity when receiving cervical cancer screening (developing a web page or professional counseling centers) have been discussed. Therefore, based on the findings above, we argue that socio-cultural approaches to respond to the closed sexual culture of unmarried young women could play an essential role in increasing their participation in cervical cancer screening.

The limited knowledge level on cervical cancer screening was prominent in both quantitative and qualitative methods. Moreover, the knowledge level of those who had been screened for cervical cancer was unsatisfactory. These findings were consistent with other studies that targeted university students in other countries [17, 20]. Seriously, in the focus groups, some participants who had never been screened confused cervical cancer screening with HPV vaccination and said that they had not learned or heard about cervical cancer or screening in high school. This might be closely related to Korea’s high school educational environment, where sexuality education issues are relatively marginalized by the university entrance exam-oriented educational system [21]. Thus, even if university students begin to be more actively exposed to sexual experience, most do not recognize the importance of sexual health and rely primarily on mass media and their peers for the information they need [11]. Young individuals are subject to normative influences from their peers, especially sex-related issues [17]. In this context, previous studies used peer educators to disseminate information on sexual health, such as sexually transmitted diseases and AIDS and reported its effectiveness in improving sexual health behaviors [22–25]. Similarly, in our study, the use of peer educators was discussed as a strategy to improve the knowledge level of cervical cancer screening.

Other strategies to improve the level of knowledge included online communication and information channels, with an easy and straightforward description of contents and detailed information for initial screening. As online social networking sites have been commonly used for sexual health promotion among the young population, Sun et al. (2017) reported that education on safe sexual health using Facebook yielded significantly positive results in university students [22]. Lee et al. (2014) reported that 23% of young women who had not undergone cervical cancer screening were screened through interventions that used mobile phone text messages [26]. The use of easy and straightforward descriptions might explain health literacy and the understanding of health information. A high level of health literacy was significantly related to health promotion behaviors such as HPV vaccination and general health among university students [27, 28]. Moreover, some participants in our study suggested that more detailed information on cervical cancer screening procedure and cervical cancer should be provided at a young age. Typically, for young women, perceived susceptibility and threat of cervical cancer are pretty low because health is not a major concern [11, 17]. Therefore, to increase knowledge level, various educational strategies, including educational delivery media and content that can be sensitive to the culture of young women, should be developed.

All three groups highlighted psychological burden as one of the main barriers to cervical cancer screening and a practical burden. Previous studies reported that, regardless of age, most women experience emotional discomfort due to the exposure of private part for vaginal examination, aversion to male doctors and practical discomfort related to the time consumed in-clinic visits [17, 20]. In particular, all participants in our study mentioned the fear of male doctors performing cervical cancer screening as a cause of psychological burden. The participants suggested alternative methods with minor discomforts, such as blood tests, to alleviate the psychological burden, including anxiety. In addition to this, focus groups presented the following methods as more active ways to overcome psychological burden: 1) providing detailed information that, even if they are young and have no symptoms, all women with sexual experience have a risk of developing cervical cancer, and 2) providing detailed information on the screening procedure.

Our study had some limitations. First, with the exception for snowball sampling, the study participants’ recruitment was mainly conducted at a university in a metropolitan area, and thus there are limitations in generalizing the results. However, the participants’ homogeneity would have allowed us to conduct in-depth interviews. More importantly, as students studying nursing and medicine were excluded, the results may have contributed significantly to understanding the perceptions of female university students who have fewer opportunities to access health education. Second, the sample size calculation for the quantitative survey was not considered. This was mainly because the quantitative survey was focused on obtaining accurate sex-related information (which may be sensitive in Korea) and enhancing participant understanding before the FGIs. Finally, because of social desirability, the participants may have come up with ideas that match the researcher’s expectations. To address this issue, we employed an experienced moderator, a registered nurse, who richly drew upon details and the opinions held by the participants.

## Conclusions

In conclusion, compared with the rapid increase in female university students’ sexual experience, they were neither sufficiently oriented nor educated on sexual health regarding cervical cancer screening and cervical cancer. As supported by this, a low level of knowledge on cervical cancer screening was evident in our results, especially in those who had not been screened. Moreover, the negative social perception of unmarried female university students’ sexual activities had a great influence as a specific barrier to screening and a low level of knowledge. Psychological and physical burdens related to the test procedure also emerged, but these findings may be exaggerated due to the limited knowledge level of the screening procedure. Based on the strategies identified to overcome these barriers, we suggest establishing socio-cultural approaches to change the negative social perception of unmarried young women’s sexual activities and educational interventions sensitive to their culture.
